# Molecular Imaging of Diabetic Foot Infections: New Tools for Old Questions

**DOI:** 10.3390/ijms20235984

**Published:** 2019-11-28

**Authors:** Camilo A. Ruiz-Bedoya, Oren Gordon, Filipa Mota, Sudhanshu Abhishek, Elizabeth W. Tucker, Alvaro A. Ordonez, Sanjay K. Jain

**Affiliations:** 1 Center for Infection and Inflammation Imaging Research, Johns Hopkins University School of Medicine, Baltimore, MD 21287, USA; cruizbe1@jhmi.edu (C.A.R.-B.); ogordon3@jhmi.edu (O.G.); fdamota2@jhmi.edu (F.M.); sabhish1@jhmi.edu (S.A.); etucker9@jhmi.edu (E.W.T.); aordone2@jhmi.edu (A.A.O.); 2 Center for Tuberculosis Research, Johns Hopkins University School of Medicine, Baltimore, MD 21287, USA; 3 Department of Pediatrics, Johns Hopkins University School of Medicine, Baltimore, MD 21287, USA; 4 Department of Anesthesiology and Critical Care Medicine, Johns Hopkins University School of Medicine, Baltimore, MD 21287, USA; 5 Division of Pediatric Critical Care, Johns Hopkins All Children’s Hospital, St. Petersburg, FL 33701, USA; 6 Russell H. Morgan Department of Radiology and Radiological Science, Johns Hopkins University School of Medicine, Baltimore, MD 21287, USA

**Keywords:** diabetes, diabetic foot infection, bacterial infections, PET imaging, SPECT imaging, radionuclides probes, MRI

## Abstract

Diabetic foot infections (DFIs) are a common, complex, and costly medical problem with increasing prevalence. Diagnosing DFIs is a clinical challenge due to the poor specificity of the available methods to accurately determine the presence of infection in these patients. However, failure to perform an opportune diagnosis and provide optimal antibiotic therapy can lead to higher morbidity for the patient, unnecessary amputations, and increased healthcare costs. Novel developments in bacteria-specific molecular imaging can provide a non-invasive assessment of the infection site to support diagnosis, determine the extension and location of the infection, guide the selection of antibiotics, and monitor the response to treatment. This is a review of recent research in molecular imaging of infections in the context of DFI. We summarize different clinical and preclinical methods and the translational implications aimed to improve the care of patients with DFI.

## 1. Introduction

The burden of diabetes has steadily increased across the globe in the last 25 years. In 2017, it was responsible for 2.71% of disability-adjusted life years and 2.45% of deaths worldwide [1]. While diabetes is a systemic disease, diabetic foot infections (DFIs) are the most common complication, accounting for ~25% of hospitalizations in diabetic patients and leading to increased healthcare costs [2,3]. DFIs increase premature mortality and are the predominant cause of non-traumatic limb amputations, which have a significant impact on the quality of life of more than 200,000 diabetic patients that suffer an amputation every year [4]. Multiple factors, including poor glycemic control, immunosuppression, peripheral vasculopathy, and peripheral neuropathy, contribute to bacterial colonization and proliferation within diabetic foot ulcers (DFUs). These are mostly polymicrobial infections that usually spread from the soft tissue to the adjacent structures such as bone.

Despite advances in clinical practice in the last decades, the diagnosis and management of DFIs remain a challenge. Traditional methods to diagnose DFIs (e.g., microbial cultures) rely on clinical samples that are prone to contamination with normal microflora, sampling bias, and difficulties in identifying fastidious microorganisms [5]. Invasive tissue sampling is also usually limited to a single time-point and a single location and does not provide a comprehensive perspective of all the spatial and temporal heterogeneity of the infection process. This may result in suboptimal management [6]. Conversely, failure to differentiate true infection from inflammation can lead to unnecessary antibiotic overuse. Thus, there is an unfulfilled clinical need for non-invasive tools that can rapidly diagnose or exclude the infection and determine its depth and extension. Similarly, new tools are needed to non-invasively monitor and predict therapeutic response. Molecular imaging modalities could play a major role in answering these critical questions [7]. Therefore, this review provides an overview of the current challenges of DFIs and the opportunities for molecular imaging to understand its pathogenesis, clarify diagnostic uncertainty, and enhance antimicrobial treatment, with an emphasis on novel imaging technologies that may address some of the fundamental questions from bench-to-bedside. Positron emission tomography (PET) is considered one of the most sensitive molecular imaging technologies, and therefore, will be highlighted in this article, although other technologies such as single-photon emission computed tomography (SPECT), magnetic resonance imaging (MRI), and optical imaging also hold substantial promise.

## 2. Challenges in the Diagnosis and Treatment of DFIs

### 2.1. Is There An Infection?

**Figure 1:**
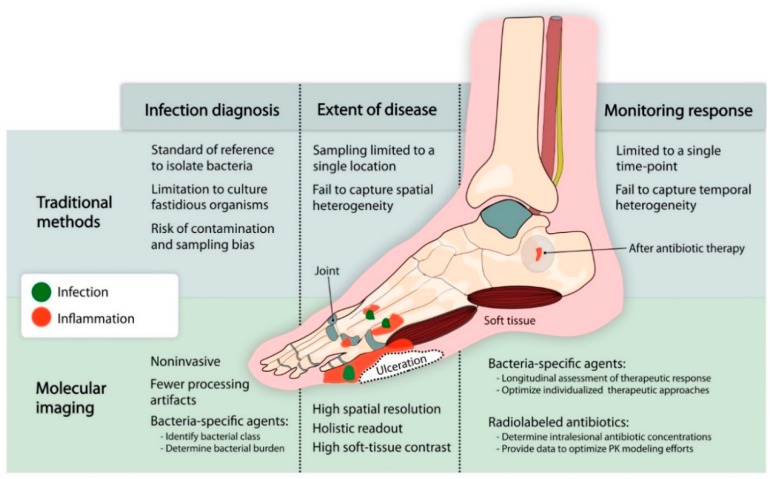
Limitations of traditional methods and opportunities for molecular imaging to diagnose and monitor diabetic foot infection (DFI). Diabetic foot infections have traditionally been evaluated using invasive techniques (e.g., microbiological cultures, bone biopsy) that can miss the site of infection and do not reflect the heterogeneity of the disease, or by non-specific methods (e.g., serum biomarkers, plain radiographs, etc.) that cannot correctly differentiate between infection and sterile inflammation (e.g., Charcot’s neuroarthropathy). Novel developments in molecular imaging provide new non-invasive tools that can accurately differentiate infection from inflammation, determine the location and extent of the disease, characterize the conditions of the microenvironment at the infection site, and monitor the response to antibiotic therapy.

When faced with a diabetic patient with a possible DFI, clinicians face a complex diagnostic scenario (Figure 1). Current diagnostic workflows rely more on clinical manifestations than on microbiological (e.g., positive wound cultures), histopathological, serum inflammatory markers or imaging readouts [8]. Due to the high risk of false-positive and negative results, the growth of bacteria in a culture obtained from a wound sample or the presence of high levels of serum inflammatory markers (e.g., erythrocyte sedimentation rate (ESR), C-reactive protein, and procalcitonin) in the absence of clinical manifestations is usually not enough to make a definite diagnosis [9,10,11]. Direct tissue sampling for histopathological analysis can also be falsely negative when the site of infection is missed due to a sampling error [9,12].

Anatomical imaging (e.g., plain radiograph, MRI, computed tomography (CT), and ultrasonography) can provide valuable information on the extent of the tissue damage. However, these non-specific tissue changes are mostly due to the inflammatory response and occur late in the disease process compared to the early molecular events occurring at the site of infection. Molecular imaging tools such as SPECT or PET provide a comprehensive method to measure molecular pathways and can be used in conjunction with CT and MRI to provide an anatomical reference. The most widely used clinical PET imaging agent is ^18^F-fluorodeoxyglucose (^18^F-FDG), a radiolabeled analog of glucose that accumulates in areas with increased glucose metabolism due to infection and inflammation. Similarly, radiolabeled leukocyte scintigraphy also detects areas of infection and inflammation. While both technologies are highly sensitive, provide a high negative predictive value, and play an important role in determining the extent and location of the infection (see additional information below), they are unable to discriminate true infection from sterile inflammatory processes such as Charcot’s neuroarthropathy (CN).

There is an urgent need for new technologies that can provide a faster and reliable characterization of the pathogen, its susceptibility and response to therapy, especially in patients with DFI. Pathogen-specific molecular imaging based on PET and SPECT technologies is an emerging field with promising opportunities for visualizing infectious processes. Radiolabeled agents that selectively accumulate/bind to prokaryotes can be visualized with PET/SPECT to identify and characterize the infection site. However, these agents require critical characteristics of tissue penetration, sensitivity, and specificity, among other factors, to have an optimal performance in the setting of DFIs (Table 1). Based on these requirements, radiolabeled small molecules and drug-like organic small molecules that accumulate in bacteria hold promise. Ordonez et al. performed an in silico screening of 961 small molecules with these characteristics, finding several favorable compounds for the most representative classes of bacterial pathogens [13]. Notably, D-sorbitol accumulated extensively in *Enterobacteriales* order of Gram-negative bacteria, *para*-aminobenzoic acid (PABA) accumulated in all bacterial species, and D-mannitol accumulated selectively in Gram-positive and Gram-negative bacteria, but not in mycobacteria. Several of these bacteria-specific molecules have been radiolabeled with different high-energy emission radionuclides [14]. PABA is selectively incorporated into all types of bacteria as part of the folate pathway, and it is significantly accumulated in all growth phases, including in dormant bacteria [13]. ^11^C and ^18^F-labeled analogs of PABA were able to accurately differentiate infection from inflammation using PET imaging in mouse and rat models of *Staphylococcus aureus* and *Escherichia coli* myositis [14,15]. Targeting the same folate pathway, a PET agent based on the broad-spectrum antibiotic trimethoprim, ^18^F-fluoropropyl-trimethoprim (^18^F-FPTMP) has shown promising results in animal models of infection [16]. Radiolabeled D-amino acids, nucleoside analogs, siderophores, and antimicrobial peptides have also been successfully developed and are able to detect infections with high specificity [17,18,19,20,21,22].

Multiple sugar analogs that selectively target prokaryote-specific metabolism have been radiolabeled for PET imaging of bacterial infections. For example, ^18^F-labeled maltohexaose and 6″-^18^F-fluoromaltotriose are incorporated into bacteria using the maltodextrin/maltose transporter and accumulate in a broad range of Gram-positive and Gram-negative pathogens [23,24]. Similarly, ^18^F-fluorodeoxysorbitol (^18^F-FDS) is a radiolabeled analog of sorbitol that selectively accumulates (~1000-fold higher than mammalian cells) in *Enterobacteriales* group of bacteria [25], which are present in ~40% of DFIs [11]. With the advantage of a rapid synthesis based on the chemical reduction of ^18^F-FDG in ~30 min, ^18^F-FDS has been reported to accurately differentiate infection from inflammation in mouse models of myositis, brain abscess, and pneumonia [25]. ^18^F-FDS has also been evaluated in healthy volunteers and infected patients with promising results [26,27]. ^18^F-FDS PET was safe and well-tolerated in all subjects evaluated, with optimal imaging 60–120 min after tracer injection [26]. In infected patients, ^18^F-FDS PET could detect foci of infections in multiple sites, including musculoskeletal infections. Additionally, ^18^F-FDS PET was also able to monitor response to antibiotic treatments with a decrease in PET signal correlating with clinical improvement [27]. A recent report by Cheng et al. claimed ^18^F-FDS accumulation at the site of a pituitary spindle cell carcinoma (SUV 1.49) in a 33-year-old patient [28]. However, imaging in this patient was performed 5 min after ^18^F-FDS injection [29], and blood pool effect, i.e., capillary leak at the site of the tumor, is likely responsible for this finding [30]. In fact, dynamic ^18^F-FDS PET studies in mice with U87MG brain tumors demonstrate some initial signal, which, however, dissipated 60–120 min after tracer injection [25]. This is consistent with the lack of ^18^F-FDS uptake by normal or cancerous mammalian cells reported by Li et al. and Weinstein and Ordonez et al. [25,31]. Importantly, there is no selective transporter for ^18^F-FDS entry into mammalian cells, and the substitution of the hydroxyl group by fluorine at the C2-position abrogates the recognition by the mammalian sorbitol dehydrogenase [32]. This suggests that the results presented by Cheng et al. are consistent with a non-specific blood pool effect; that is, capillary leak at the site of inflammation or tumor [30]. ^18^F-FDS is also the first bacteria-specific PET agent to target a distinct bacterial class and could be used to non-invasively identify the causative pathogen in DFIs. Recently, an MRI technique utilizing endogenous (chemical exchange saturation transfer (CEST)) contrast has also been developed to specifically detect bacterial infections with promising results, demonstrated in an animal model of *S. aureus* brain infection [33].

### 2.2. Which Pathogen(s) Are Causing the Infection?

Once the infection is clinically suspected, identifying the pathogen(s) causing the infection and their antibiotic susceptibility is highly important to select the appropriate antimicrobial therapy, especially when multidrug-resistant organisms (MDROs) are likely to be present. In DFI, antimicrobial therapy is selected based on severity and risk factors. Newly infected DFIs are usually polymicrobial, while chronically infected foot ulcers tend to be monomicrobial [34]. In monomicrobial infections, the most common aerobic Gram-positive bacteria is *S. aureus*, especially in cases of osteomyelitis where it is the most frequently isolated single pathogen [35,36]. Soft tissue infections are caused mainly by *S. aureus* or beta-hemolytic streptococci. Chronic ulcers that are treated with antibiotic therapy are caused mainly by Gram-negative bacilli (e.g., *Pseudomonas aeruginosa*, *E. coli, Klebsiella pneumoniae*, *Proteus* spp.), *S. aureus*, or streptococci. Osteomyelitis and gangrene associated with DFI can be polymicrobial, including anaerobes (*Bacteroides fragilis* group and *Clostridium* spp.), Gram-negative bacilli, and *S. aureus* [37,38]. There is a significant increase in the prevalence of highly resistant organisms, mainly Gram-negative bacteria that produce extended-spectrum beta-lactamase (ESBL) or carbapenemases, and methicillin-resistant *S. aureus* (MRSA) [39,40,41,42]. Despite the improvements in microbiological techniques during the past decade [43], accurate diagnosis still requires an experienced multidisciplinary team capable of collecting and analyzing the specimen from the bone or soft tissue with low risk of contamination and sampling error.

In cases where performing direct tissue sampling has a high risk of contamination or is restricted due to the patient’s comorbidities, bacteria-specific molecular imaging could serve as a diagnostic tool to identify the class of bacteria present at the infection site, and thereby support the selection of appropriate empiric antimicrobial therapy. An agent like ^18^F-FDS that selectively targets the *Enterobacteriales* group of bacteria could be used in conjunction with a broad-spectrum imaging agent (e.g., ^11^C-PABA) to determine the presence of infection and differentiate between Gram-positive and Gram-negative bacteria.

### 2.3. Where Is the Infection and Where Does It Extend to?

Determining the location of the infection and bone involvement is a key step to ensure accurate treatment and follow up. However, osteomyelitis is difficult to diagnose and is considerably harder to treat once the infection is established [43]. The combination of bone culture and histology is currently the most definitive available method of diagnosing osteomyelitis [11]. However, bone histology may not show bone inflammation in case of sampling error (osteomyelitis has a patchy distribution in the bone) or difficulty interpreting the histology [44]. Meyr et al. showed an evident lack of diagnostic agreement between pathologists for osteomyelitis in DFI, reporting a κ coefficient of 0.31, far below the average level of a reference standard test [45]. Overall, bone biopsies are not practical, have a high error probability, fail to capture the temporal and anatomical heterogeneity of the infection site, and should be considered only when it is difficult to determine the causative pathogen or its antibiotic susceptibility using different methods [11].

Several imaging tests for the diagnosis of osteomyelitis in DFI are available. Historically, plain radiography of the foot has been widely used in cases of suspected osteomyelitis. The appearance of regional osteopenia, periostitis or periosteal reaction, cortical loss or focal lysis, endosteal scalloping, change of trabecular bone architecture (demineralization), peripheral sclerosis, or/and periosteal new bone formation is highly suggestive of osteomyelitis and is usually enough to validate the diagnosis [11]. However, these findings may not be seen in the early phases of osteomyelitis, which require a high degree of bone demineralization to be detectable (>30%), and plain radiography is not proficient in differentiating between osteomyelitis, soft tissue infection, and CN [46,47]. Also, a plain radiograph is based on a two-dimensional visualization of the anatomy, resulting in the superimposition of structures, which, added to the poor image quality, leads to poor inter-observer reproducibility [48]. Three-dimensional CT imaging is more sensitive than a plain radiograph to visualize the sequestrum formation (necrotic cortical or medullary bone), involucrum (cortical collar of new bone), cloaca (draining sinus), and to evaluate the integrity of trabecular and cortical bone [47,49].

MRI enables the recognition of bone edema with accurate anatomical localization as early as 3–5 days from the onset of infection and is considered the primary imaging modality in DFIs with high spatial resolution and soft tissue contrast [50]. MRI has high sensitivity for the diagnosis of osteomyelitis, showing edematous changes with high signal on T2 and STIR sequences and decreased bone marrow signal on T1-weighted sequence [50]. Gadolinium contrast T1 imaging in patients with normal renal function is a highly precise method to establish the extent of infection, as it is also able to detect the presence of secondary signs of osteomyelitis such as ulcers, sinus tracks, cellulitis, tenosynovitis, and abscesses [49]. However, these findings frequently overlap with other conditions such as trauma, healing osteonecrosis, gout, ischemia, recent surgery, and, most importantly, acute CN [51,52]. While there are some features that could help differentiate between osteomyelitis and CN (focal vs. multiple-bone involvement, location, the presence of foot deformity and secondary signs of infection), this differential diagnosis is a frequent clinical challenge in diabetic patients [52].

The traditional nuclear medicine approach of performing a triple phase bone scan for suspected osteomyelitis with radioactive ^99m^-technetium-methylene diphosphonate has been largely replaced by radiolabeled leukocyte imaging, which has been extensively used in the clinical diagnosis of DFI. SPECT or planar imaging with radiolabeled autologous white blood cells (primarily neutrophils) are able to accurately localize the infection site. The 2012 American College of Radiology guidelines suggest the use of leukocyte scans as the first-line nuclear medicine study to evaluate patients with DFI [53]. The advantages and characteristics of this technique have been previously reviewed in comprehensive publications by Palestro et al. [54,55,56]. SPECT/CT imaging provides some advantages from planar scintigraphy, such as improved spatial resolution, better soft tissue contrast, and anatomical localization of the infection. Unfortunately, heterogeneity in the imaging protocols and interpretation, added to the poor specificity to differentiate infection from inflammation are major limitations [51,57].

Clinical PET provides a non-invasive, three-dimensional image with higher spatiotemporal resolution and high sensitivity compared to SPECT, ultrasound, MRI, and optical imaging [7]. ^18^F-FDG is an analog of glucose that is widely used as a diagnostic PET radiotracer for clinical oncology, neurology, cardiology, and even in infectious diseases [11]. Because of its accumulation in immune, bacterial, and tumoral cells, ^18^F-FDG PET is not able to differentiate between an infection (e.g., osteomyelitis), neoplasia, or sterile inflammation (e.g., CN). Furthermore, the uptake of glucose can remain impaired for 3–4 months after surgery or trauma [54]. ^18^F-FDG PET is useful in the diagnosis of osteomyelitis in patients with diabetic foot, especially because of its high spatial–temporal resolution (needed to detect infections in small bones [58]), complete view of the whole body, three-dimensional anatomic localization, and the ability to provide fast results with low radiation exposure [11,59]. Studies published in the last two decades have described the variable performance of ^18^F-FDG PET in the assessment of DFI with a wide range of sensitivity and specificity [57,60]. Differences in patient populations, co-existing conditions such as hyperglycemia and peripheral artery diseases (PAD), and antibiotic usage make direct comparisons between multiple studies challenging [61,62]. While some preliminary studies suggest that hyperglycemia does not affect the diagnostic performance of ^18^F-FDG, further validation is still required [63,64]. To validate the role of ^18^F-FDG PET in the evaluation of osteomyelitis in DFIs, large clinical studies are needed with optimized protocols for imaging and patient selection, taking into account glycemia, PAD, infection characteristics, clinical signs, etc. The diagnostic performance of ^18^F-FDG PET should also be evaluated separately for osteomyelitis vs. soft tissue infection and osteomyelitis vs. CN. Finally, the increasing availability of PET/MRI can lead to combined diagnostic algorithms that could improve sensitivity and specificity [65].

In addition to all the advantages of PET/SPECT imaging, combined with CT or MRI for anatomical co-registration, the use of bacteria-specific imaging agents has the potential of providing information on the infection location and extension with both high sensitivity and specificity. SPECT/CT studies with the radiolabeled antimicrobial peptide ^99m^Tc-UBI 29–41 have been performed to evaluate musculoskeletal infection with promising results [66]. More recently, the PET agent ^68^Ga-NOTA-UBI was used for imaging DFI in a small patient sample [67]. Similar studies have evaluated ^99m^Tc-radiolabeled antibiotics, which suggest they could be used for the detection of osteomyelitis [68]. However, some radiolabeled antibiotics and antimicrobial peptides have been shown to have poor specificity [69,70].

## 3. Is the Antibiotic Penetrating the Infection Site?

Antibiotic management starts with the selection of empiric coverage. Mild-to-moderate infections, in patients with no prior antimicrobial exposure, are initially treated with antibiotics targeting aerobic Gram-positive cocci [34,71]. For most severe infections, or for patients with increased risk of resistant bacteria, broad-spectrum antibiotics are initiated covering Gram-positive, Gram-negative, and anaerobic bacteria [34,71,72]. Definitive therapy is based on results of an appropriately obtained culture, sensitivity testing of a wound and/or deeper specimen, and the patient’s clinical response to the empiric regimen. Antibiotic tissue penetration, particularly accumulation within the bone, is a major consideration when choosing therapy for DFI. Various antibiotics show significantly different penetration to tissue [73,74] and consideration should also be given to the nature of the disease, with reduced tissue blood supply due to PAD and atherosclerosis [8,11]. The duration of antibiotic treatment is generally based on the degree of severity of infection. Short antibiotic courses for soft tissue infection are generally 1–2 weeks for mild infections and 2–3 weeks for moderate-to-severe infections. When osteomyelitis is diagnosed, the duration of antibiotic therapy relays on the nature of the surgical intervention, if performed. When a radical resection leaves no remaining infected tissue, antibiotic therapy is usually short (2–5 days). When there is persistent infected or necrotic bone, antibiotic treatment is prolonged (≥4 weeks) [8,72,75]. If there is no clinical improvement following antibiotics, it is recommended to perform another invasive procedure to get cultures to direct treatment. The International Working Group on the Diabetic Foot (IWGDF) recommendation is not to extend antibiotic treatment over 6 weeks [11]. However, other than repeating biopsy, there is currently no validated test to evaluate for on-going infection during the treatment course of DFI, and even this invasive method is prone to errors. This is the reason that, in many instances, antibiotic treatment is extended further than 6 weeks. Thus, there is an eminent unmet clinical need for DFI non-invasive diagnostics that can monitor response to treatment, particularly when the bone is involved. Knowing in real-time the extent of bone involvement may facilitate decreasing the need for extensive surgical interventions, including amputations.

As discussed above, penetration to tissue, particularly to the bone, is a major consideration when choosing antibiotics. Current understanding is based on data from a limited number of animal and human studies [74]. Importantly, direct measurements are generally limited to sampling a single, accessible lesion, at a single time point, in a particular patient population. Since infections can occur at different sites within the same organ or throughout the body, traditional approaches (e.g., biopsy or resection) at a single (or few) sites may not provide complete information about the underlying pathology, and thus can lead to sampling bias. This is particularly important in DFI, where the milieu at the infection site can alter blood supply and local drug penetration. Molecular imaging using radiolabeled antibiotics can be used to non-invasively quantify drug penetration into the sites of infection and could potentially inform on bone penetration in a personalized manner. Several antibiotics have been radiolabeled and their in vivo biodistribution and penetration into the brain and areas of pulmonary infections characterized in animal models and humans [7,76,77,78,79,80,81]. For example, in tuberculous (TB) meningitis, the most devastating form of TB with high mortality, PET imaging of radiolabeled ^11^C-rifampin showed that the drug has limited and spatially heterogeneous brain penetration that rapidly decreases as early as two weeks of TB treatment [81]. Quinolones are amongst the class of antibiotics with the highest bone penetration and several fluoroquinolones are used to treat bone infections. In healthy volunteers, ^18^F-ciprofloxacin showed to be safe and useful to determine its pharmacokinetics using PET imaging [78]. To the best of our knowledge, PET imaging with radiolabeled antibiotics has not yet been explored in the context of DFI. Since infections and other conditions can alter drug metabolism, and the infection-associated inflammatory responses and PAD can hinder antibiotic penetration, we propose that PET imaging with radiolabeled antibiotics could be used in patients with DFI to inform on drug penetration to the site of the infection, which would allow a more effective treatment.

### Can We Use Molecular Imaging to Monitor Response to Treatment?

One of the key challenges in the management of DFI is monitoring the response to antimicrobial therapy and when to stop it [11]. Several imaging techniques have been applied for detecting infection and monitoring antibiotic response in DFI, including plain radiographs, ^99m^Tc-labeled bone scans, labeled white blood cells, ^18^F-FDG-PET/CT, and MRI [82,83,84,85,86,87]. Among these, ^18^F-FDG-PET/CT and MRI show particularly promising results [88]. Nawaz et al. prospectively compared MRI to ^18^F-FDG-PET in a blinded study, showing a similar performance of the two modalities [89]. ^18^F-FDG-PET and bone scan images return to baseline after successful treatment, but the timing for this resolution varies considerably between patients [88,90,91].

**Figure 2:**
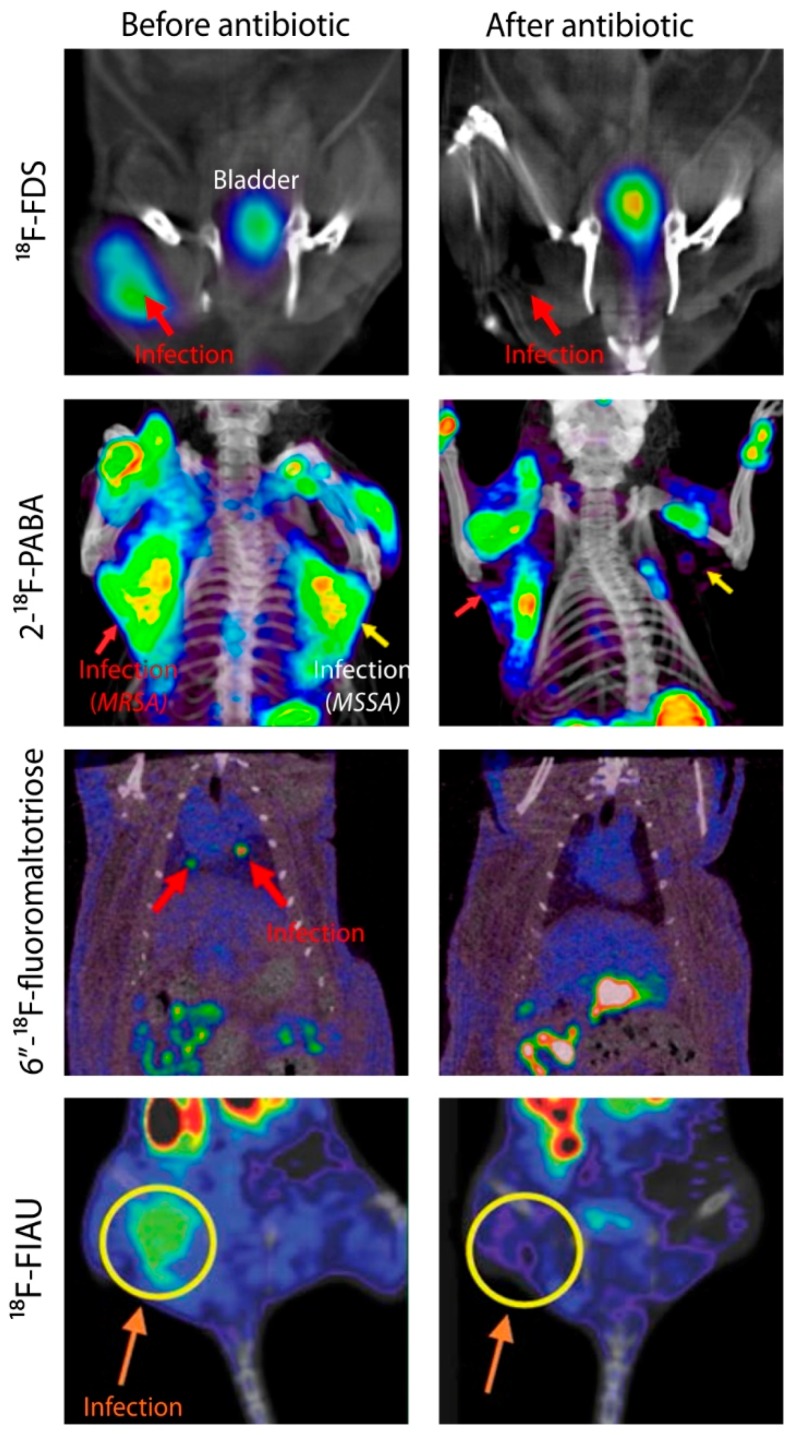
Monitoring antibiotic efficacy using bacteria-specific PET agents. Animal models of infection were used to determine the efficacy of bacteria-specific PET imaging agents to monitor antibiotic treatment. Methicillin-susceptible *Staphylococcus aureus* (MSSA); methicillin-resistant *S. aureus* (MRSA). ^18^F-Fluorodeoxysorbitol (^18^F-FDS) PET/CT images were adapted with permission from Weinstein and Ordonez et al. (reprinted with permission by the American Association for the Advancement of Science from Weinstein and Ordonez et al. 2014 [25]). 2-^18^F-*p*-Aminobezoic Acid (2-^18^F-PABA) PET/CT images were adapted with permission from Zhang and Ordonez et al. [15]; 6″-^18^F-Fluoromaltotriose PET/CT images were adapted with permission from Gowrishankar et al. [23]; this research was originally published in JNM. Gowrishankar et al. Specific Imaging of Bacterial Infection Using 6″-^18^F-Fluoromaltotriose: A Second-Generation PET Tracer Targeting the Maltodextrin Transporter in Bacteria. 2017; 58: 1679–1684. ©SNMMI. ^18^F-FIAU PET/CT images were adapted with permission from Rajamani et al. [92].

In the last five years, multiple bacteria-specific PET tracers have been used to monitor antimicrobial treatment efficacy in preclinical models (Figure 2). This is based on the highly conserved uptake mechanisms for several metabolism-based bacteria-specific tracers in development. In fact, scores of random clinical isolates, including multidrug-resistant isolates have been shown to maintain high tracer uptake [13,25]. Weinstein and Ordonez et al. showed a rapid assessment of therapeutic efficacy and pathogen antibiotic susceptibility using a sequential ^18^F-FDS PET scan before and after antibiotic therapy. ^18^F-FDS PET signal intensity disappeared in animals infected with drug-susceptible *E. coli* but persisted in animals infected with ESBL-producing *E. coli* after 24 h of antibiotic treatment with Ceftriaxone [25]. Zhang and Ordonez et al. presented similar results in rats infected with methicillin-resistant *S. aureus* (MRSA) and methicillin-susceptible *S. aureus* (MSSA). Although 2-^18^F-PABA PET has a high background activity, the signal intensity decreased in animals infected with MSSA, but persisted in animals infected with MRSA after 20 h of Oxacillin treatment [15]. Rajamani et al. presented a rapid (4 h) decrease in the ^18^F-fialuridine (FIAU) PET signal intensity of Ciprofloxacin-treated animals infected with *P. aeruginosa* [92]. Gowrishankar et al. showed that the 6″-^18^F-fluoromaltotriose PET signal disappeared after one month of antimicrobial therapy in rats with botryomycosis-like lesions in the lung due to *S. aureus* [59]. Nibbering et al. also showed that ^99m^Tc-UBI 29-41 allows monitoring of antimicrobial efficacy in both sensitive and resistant *S. aureus* strains [93]. It should be noted that clinically relevant acute bacterial infections have high bacterial burdens (8.3 Log_10_ CFU/mL) [94] and can be as large as several centimeters in diameter, with volumes of hundreds of millimeters. Similarly, bacterial burdens associated with chronic bacterial infections such as TB can be high (7–9 Log_10_ CFU/mL). Since many bacteria-specific tracers allow detection of as few as 6 Log_10_ CFU/mL, this is indeed a promising threshold for many clinically important bacterial infections. Given that some of these tracers (e.g., ^18^F-FDS, ^99m^Tc-UBI 29-41, ^68^Ga-NOTA-UBI) are now being evaluated in humans, bacteria-specific molecular imaging could provide a unique opportunity to monitor the treatment of patients with DFIs. However, the sensitivity of these agents still needs to be further evaluated and validated in larger clinical trials, especially in chronic infections with a lower bacterial burden.

## 4. Could Molecular Imaging Evaluate Microvascular Complications in DFI?

The presence of clinically significant lower limb ischemia makes both the diagnosis and treatment of DFIs considerably more difficult [11]. In the diabetic foot, PAD presents with accelerated endothelial dysfunction and capillary microangiopathy, resulting in poor tissue perfusion and ischemia, ultimately leading to ulceration and impaired wound healing. Critical limb ischemia is associated with a high incidence of lower limb amputation, and premature mortality [11]. Although revascularization techniques have been shown to improve rates of wound healing and overall limb salvage outcomes, clinical techniques routinely used to assess PAD are limited and do not offer an accurate representation of microvascular flow at the site of disease.

Assessment of PAD in patients with foot ulceration often begins with a thorough history of symptoms suggestive of PAD, followed by the use of imaging techniques. Doppler-measured ankle-branchial pressure index (ABI) is a non-invasive technique useful in the assessment of PAD; however, it has been found unreliable in patients with diabetes who may report falsely elevated pressure due to calcification [95,96]. Similarly, toe pressure and continuous Doppler waveform assessment have also been found unreliable in diabetic patients [97]. Once a diagnosis of PAD has been established, it is important to obtain anatomical information regarding the extent of ischemia, as well as local perfusion. Transcutaneous oxygen tension (TcPO_2_) indirectly reports on perfusion by measuring the transfer of oxygen molecules to the skin surface. Although more sensitive than ABI in patients with diabetes, TcPO_2_ is affected by the metabolic demands of the tissue, thus being unreliable in patients with an active infection [98,99]. Fluorescence angiography using indocyanine green dye is not a routine clinical procedure but has been used in patients with critical limb ischemia, including patients with diabetic foot ulcerations (status of infection not reported), to assess perfusion and response to vascularization [100].

Novel optical techniques for imaging microcirculation in the diabetic foot include laser doppler perfusion imaging, laser speckle contrast imaging, photoacoustic imaging, and hyperspectral imaging. Although promising, these techniques are still being evaluated in clinical scenarios [101]. Similar to using PET and SPECT imaging to assess brain and myocardial perfusion, a molecular imaging approach to evaluate microvascular perfusion in the diabetic foot would allow a clear delineation of the ischemic regions and could potentially be used to monitor treatment response to revascularization techniques.

A pilot study with ^99m^Tc-tetrofosmin was performed in diabetic patients with critical limb ischemia. SPECT/CT imaging demonstrated qualitative and quantitative differences in microvascular foot perfusion between the affected patients and healthy controls at rest. In diabetic patients, there was no correlation between the uptake of ^99m^Tc-tetrofosmin and ABI, suggesting that SPECT/CT perfusion imaging might be a more sensitive technique [102]. This study highlights the advantages of molecular imaging in assessing the pathophysiology of diabetic foot infections and the potential for other radiotracers to be evaluated in this clinical scenario. For example, ^99m^Tc-sestamibi, also a perfusion radiotracer, has been used to assess lower limb perfusion [103]. It should be noted, however, that although SPECT imaging of microvascular perfusion has been assessed in the context of the diabetic foot, it does not provide information regarding the infection itself.

## 5. Conclusions

DFI and its complications represent a frequent clinical challenge. Peripheral neuropathy-compromised immunity and poor vascularization lead to infection and inflammatory response. Key issues regarding diagnosis of infection, its location/extension, type of causative pathogen, and response to treatment are still unresolved. Delays in making an accurate diagnosis can lead to increased complications for the patient, including amputation. While molecular imaging based on non-specific agents has been a powerful tool for the last couple of decades to aid in the diagnosis and management of DFI, specific methods are required. We believe it is time to face the old questions of DFIs with new tools, harnessing the potential of bacteria-specific imaging to provide valuable information that improves diagnosis, gain a better understanding of the disease processes, optimize antibiotic therapies, and improve patient care.

## Figures and Tables

**Table 1:** Looking for the magic bullet: Desirable properties of an ideal bacteria-specific PET/SPECT imaging agent for DFIs. (Adapted from [14].)

Tissue Penetration	High tissue penetration in areas with reduced vascular supply and heterogeneous infection conditions.
Sensitive	High target-to-background signal ratio. Low limit of detection (≤10^5^ colony-forming units (CFUs)) [7].
Specific	Bacterial accumulation in both susceptible and drug-resistant organisms in different growth phases. Capable of differentiating between bacterial infection and sterile inflammation.
Specific for Gram-positive bacteria	Selective accumulation within Gram-positive pathogens. Capable of differentiating between Gram-positive and Gram-negative infections and sterile inflammation.
Quantitative	Signal proportional to the bacterial burden.
Stable	Chemically stable in blood. Low degradation of the agent by the host.
Safe	Acceptable radiation dose and repeat injection feasible without toxicity.
Manufacturable	Good manufacturing practice (GMP) production line with available PET/SPECT radioisotopes at a reasonable expense.

## Abbreviations

^11^C	Carbon-11
^18^F	Fluoride-18
^68^Ga	Gallium-68
^99m^Tc	Technetium-99m
ABI	Ankle-branchial pressure index
CN	Charcot’s neuroarthropathy
CFU	Colony-forming unit
CT	Computed tomography
DFI	Diabetic foot infection
DFU	Diabetic foot ulcer
ESBL	Extended spectrum beta-lactamase
ESR	Erythrocyte sedimentation rate
FDG	Fluorodeoxyglucose
FDS	Fluorodeoxysorbitol
FIAU	Fialuridine
FPTMP	Fluoropropyl-trimethoprim
GMP	Good manufacturing practice
MDRO	Multidrug-resistant organism
MRI	Magnetic resonance imaging
MRSA	Methicillin-resistant *Staphylococcus aureus*
MSSA	Methicillin-susceptible *Staphylococcus aureus*
NOTA	1,4,7-triazacyclononane-*N*,*N*′,*N*″-triacetic acid
PABA	*para*-Aminobenzoic acid
PAD	Peripheral artery disease
PET	Positron emission tomography
SPECT	Single-photon emission computed tomography
STIR	Short TI Inversion Recovery
TcPO_2_	Transcutaneous oxygen tension
TB	Tuberculosis/Tuberculous
UBI	Ubiquicidin
